# UV Dosage Unveils Toxic Properties of Weathered Commercial Bioplastic Bags

**DOI:** 10.1021/acs.est.3c02193

**Published:** 2023-09-26

**Authors:** Jakob Quade, Sara López-Ibáñez, Ricardo Beiras

**Affiliations:** † ECIMAT-CIM, Universidade de Vigo, Illa de Toralla, 36331 Vigo, Galicia, Spain; ‡ Facultade de Ciencias do Mar, Universidade de Vigo, 36310 Vigo, Galicia, Spain

**Keywords:** marine pollution, plastic, ecotoxicology, littoral, mesocosm, weathering, Paracentrotus lividus

## Abstract

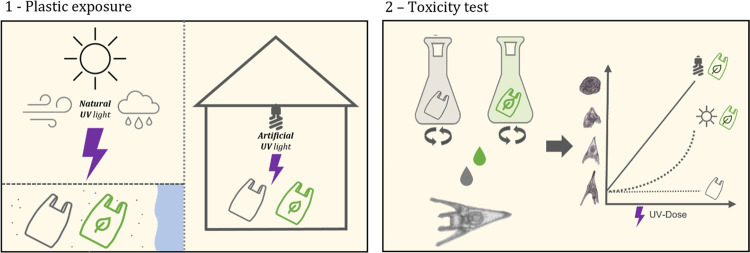

Previous studies indicated that weathered conventional plastics and bioplastics pose ecotoxicological risks. Here, the effects of artificial and natural weathering on the ecotoxicity of three compostable bags and a conventional polyethylene (PE) bag are investigated. With that aim, a 21-day artificial indoor weathering experiment featuring UV light, UV-filtered light, and darkness was run simultaneously to a 120-day outdoor littoral mesocosm exposure featuring natural light, UV-filtered light, and shaded conditions. Acute toxicity of so-weathered plastic specimens was tested *in vivo* using the sensitive *Paracentrotus lividus* sea-urchin embryo test. PE was nontoxic from the beginning and did not gain toxicity due to UV weathering. In contrast, for bioplastics, dry artificial UV weathering increased toxicity in comparison to the dark control. Weathering in outdoor mesocosm led to a rapid loss of toxic properties due to leaching in rainwater. With a higher UV dosage, a plastic-type-dependent regain of toxicity was observed, most likely driven by enhanced availability or transformation of functional additives or due to bioplastic degradation products. PE showed moderate UV absorbance, while bioplastics showed high UV absorbance. This study highlights the potential of biodegradable plastics to pose enhanced ecotoxicological risk due to weathering under environmentally relevant conditions.

## Abstract 2

The role of UV light on plastic degradation is well known; nevertheless, the associated ecotoxicity kinetics are not usually addressed. This study shows the enhanced environmental risk of compostable bioplastics depending on the UV dose received.

## Introduction

Plastic materials combine multiple advantageous properties: they are light, impermeable for most gases and liquids, and extremely durable. While those properties lead to a wide range of applications, the environmental impacts after an incorrect disposal are of concern. It is estimated that 40% of plastic products have a life span of approximately 1 month before turning into trash^1^ and, thus, increasing amounts of plastic are accumulating in the environment.^2^ A proposed solution was the development of potentially biodegradable and compostable plastics (BDCP) and bio-based plastics (BBP). These new materials, for which there is no EU law applying currently, face the challenge of possessing useful characteristics, as found in conventional oil-based plastics, while trying to avoid the associated risks.^3^ They are used for a wide range of applications, especially packaging and also textiles and electronics, and their production is continuously growing. Their market share is expected to increase from 2 to 6 million tonnes in the period from 2022 to 2027.^3^ The actual benefit of these materials was often questioned in recent years,^4−6^ and experimental evidence is compiling that bioplastics could pose ecotoxicological risks as well,^7−9^ so special caution has to be taken with these new alternatives. The adverse effects of plastics were successfully linked to chemical additives^10−13^ that provide functional traits to the plastic materials. As these additives are often not chemically bound to the polymer matrix,^14^ they are readily leachable^15−17^ and toxicity of the materials can be rapidly lost upon environmental exposure in aqueous media.^18^

UV radiation is one of the main drivers of plastic degradation in the environment.^19,20^ The highly energetical UV light radiation not only causes visible effects, such as cracks and color changes,^21^ but also impacts the materials on a molecular level, leading to the release of dicarboxylic acids and other polymer chain degradation products.^22^ To undergo photodegradation, a substance must be able to absorb light, facilitated by chromophores typically found in plastics as double bonds, aromatic groups, impurities, or photo-oxidant agents.^19,23^ The absorption of light photons by chromophore groups in the polymer chains causes random oxidation, where oxygen or free radicals bind to unsaturated links or branched chains in the polymeric matrix and eventually lead to chain scissoring and reduction of the polymer molecular weight.^24^ Photodegradation and photo-oxidation are accelerated by longer irradiation periods.^25^ Most studies addressing the effect of weathering on the toxicity of conventional plastics^26,27^ and bioplastics^8,12^ used experimental approaches based on aqueous leaching of the plastic materials, and only few studies irradiated the plastic material with a follow-up leaching step^28,29^ or investigated a time-dependent relation. This is especially interesting, as previous research showed that longer weathering periods lead to higher toxicity of plastic materials and their leachates^29^ in a not necessarily linear process.^18^

This study aimed to investigate the relation between the UV radiation dose received by the materials during weathering and the ecotoxicity of commercially available compostable and conventional plastic bags under environmentally relevant and laboratory-controlled conditions. These types of experiments are useful to classify new materials and study a priori their potential environmental risks, so as to select the ones with the properties that pose the lowest impact on the environment, knowing which factors are involved. We used the larvae of the marine model organism *Paracentrotus lividus* to assess the adverse effects of plastic leachates by measuring the sensitive sublethal endpoint larval growth inhibition.^30^ With that aim, three kinds of commercially available compostable plastics and one conventional plastic (PE) were exposed to natural and UV-filtered sunlight in a flow-through outdoor mesocosm system for up to 120 days. Simultaneously, an artificial weathering experiment was conducted indoors for 21 days in order to manipulate and control UV dose and minimize the cofactors present in outdoor conditions, such as rain, dew, and wind.

## Materials and Methods

### Description of Materials

Commercially available plastic bags with similar thickness (ca. 20 μm) but different degradability were tested. A certified home-compostable bag, hereon BIO1, two brands of industrial-compostable bags, hereon BIO2 and BIO3, and a conventional polyethylene bag, hereon PE, with the characteristics summarized in Table TS1 were purchased online. BIO1 is a green bag, certified as home compostable (OK compost HOME by TÜV Austria) and advertised as polylactide/polybutylene adipate terephthalate (PLA/PBAT) and maize starch-based. BIO2 is a light green bag; it claims compostability under industrial conditions, being certified by ASTM D6400 and the Biodegradable Products Institute, and it is advertised as made from “Bioplast,” a PLA-based polymer. BIO3 is a translucent-beige t-shirt bag, claiming compostability in industrial facilities (OK compost INDUSTRIAL by TÜV Austria) and is made from a maize starch and polymer mixture according to the producer. PE is a white, low-density polyethylene bag and was used as a non-biodegradable negative control. All three types of materials were analyzed on absorbance capability using a Jasco V650 spectrometer prior to the experimental setups. Absorbance was measured in 1 nm steps from 200 to 700 nm.

### Mesocosm Tests

**Figure 1:**
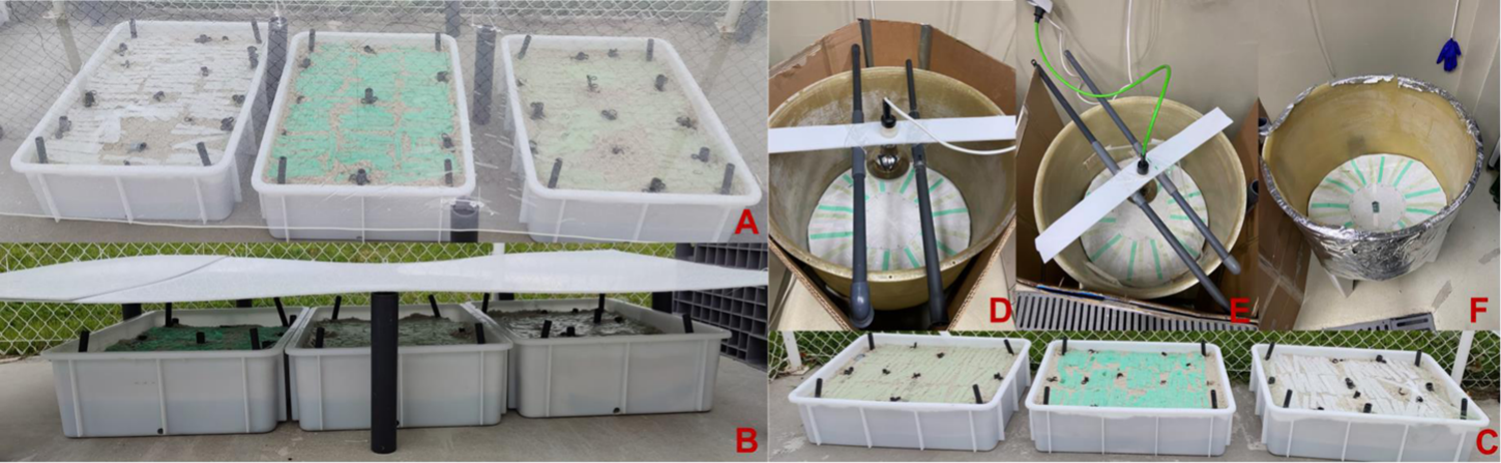
Experimental setup of the outdoor mesocosm littoral habitats: LIT_R (A), LIT_D (B), and LIT (C), and indoor artificial weathering experiment: UV_T (D), UV_F (E), and UV_D (F). The experimental conditions for the experimental setup are summarized in Table 1.

The mesocosm exposures were conducted from the 5th of April to the 3rd of August 2021 (Mesocosm-2021) and 21st of March to the 19th of July 2022 (Mesocosm-2022) at the coastal outdoor mesocosm facilities of ECIMAT-CIM (University of Vigo, Galicia, Spain), belonging to the European mesocosm network AQUACOSM-plus. The first mesocosm exposure was described in detail by Quade et al.^31^ Briefly, samples of the materials BIO1, BIO3, and PE were exposed to natural littoral conditions (LIT) in 120-L boxes, filled to the top with natural beach sand. For Mesocosm-2022, the samples of BIO1, BIO2, and PE were exposed to three different littoral simulations, each one of them featuring different amounts of solar radiation (LIT −natural–, LIT_D −shaded–, LIT_R −reduced UV−). Littoral systems (Figure 1A–C) were set up in boxes of 75x55x20 cm. Depth was reduced to 8 cm by implementing a false bottom. On top, 50 kg of sterile sand (Astralpool Silica Sand 0.4–0.8 mm) was introduced and inoculated 96 h prior to experimental start with 10% natural beach sand. LIT boxes were fully exposed to natural sunlight, LIT_D boxes were shaded from direct light using an opaque poly(vinyl chloride) (PVC) sheet, while LIT_R boxes (Table 1) were shaded using a transparent acrylic sheet to which a UV-A and UV-B filtering foil (UV-A 151-E, Reflectiv, France, UV-A transmittance = 1%) was attached (Figure S6). Sheets shadowing LIT_D and LIT_R exposures were fixed 15 cm above the sand surface. UV transmittance of the film, the acrylic sheet, and the film plus the acrylic were confirmed with a Jasco V650 spectrometer at days 0, 45, and 120 (Figure S6).

**Table 1:** Exposure Conditions in the Mesocosm Habitats (LIT, LIT_R, LIT_D) and the Three Artificial Weathering Experiments (UV_T, UV_R, UV_D)

experiment	exposure	wind	light	*T* (°C)	water
Mesocosm-2021	LIT	natural	natural unfiltered	16.9 ± 2.7	natural rain and condensation water
	LIT				
	LIT				
Mesocosm-2022	LIT	natural	natural unfiltered	21.0 ± 10.0	natural rain and condensation water
	LIT				
	LIT_R		natural filtered 1% UV	24.9 ± 12.0	splash and condensation water
	LIT_R				
	LIT_D		natural shaded	18.8 ± 4.9	splash and condensation water
	LIT_D				
artificial weathering	UV_T	no	artificial UV	50.7 ± 1.0	no
	UV_R		artificial filtered 1% UV	44.3 ± 2.3	no
	UV_D		dark	22.5 ± 1.0	no

Rectangular (2 × 17 cm^2^) specimens were cut from each plastic bag using a scalpel, avoiding edges and folds, and fixed on the sand surface with a monofilament nylon net. UV radiation was measured with Delta Ohm probes (LPUVA03 for UV-A, LPUVB03 for UV-B), and other meteorological variables were recorded by the ECIMAT weather station (Gill Maximet GMX 600). UV radiation for LIT_R was calculated using the total measured UV and the average transmittance of the shadow screen. Temperature was recorded in 30 min intervals at the sand surface in each treatment using a HOBO Pendant Data Logger (UA-002-64). For Mesocosm-2021, only environmental air temperature data were obtained. Samples from the mesocosm habitats (*n* = 10–15 when possible) were taken after 28 and 120 days of exposure in a randomized fashion from each treatment, rinsed with distilled water, and carefully cleaned with a cotton swamp to remove any biofilm and dirt without damaging the surface. Specimens were then left to dry in dark conditions at ambient temperature until constant weight was reached before further processing.

### Artificial Weathering

Additionally, an indoor UV-exposure experiment was conducted under controlled light conditions, with no interference of rain or wind. Samples were exposed to a UV lamp (OSRAM Ultra Vitalux 300W 230V E27) with an intensity of 13.6 W in the UV-A and 3 W in the UV-B spectra according to the manufacturer. The intensity was measured underneath the light source at 0 and 30 cm distance from the center by using a RAMSES ACC-UV (TriOS) radiometer. The light source was placed 36 cm above the samples in round fiberglass containers and covered with a cardboard to screen external light. Three treatments were tested: unfiltered light (UV_T), filtered light (UV_R), with a 1% UV transmittance, as used in the LIT_R treatment, and a dark control (UV_D) used as a reference (Table 1). Five plastic specimens of each material were exposed to each treatment for 21 days. Specimens were placed all at the same distance from the lamp and oriented in a radial fashion (Figure 1D–F) to ensure the same irradiation to each replicate. Total radiation and temperature were recorded throughout the experimental timeframe.

To quantify simulated aging, Gewert et al.^32^ used the mean natural irradiance of UV-A and UV-B in Europe to work out an equivalence to days of exposure in the environment. In this study, we used the total UV dose (*D*_UV_ in mJ cm^–2^) as the sum of the UV-A and UV-B radiation values (eq 1). UV dose is defined as intensity (mW cm^–2^) times the exposure time (s). The spectral ranges used to quantify UV-A and UV-B doses were as defined by ISO (2007)^33^ (UV-A = 315–400 nm, UV-B = 280–315 nm).
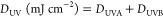
1

### Toxicity Tests

The sea-urchin embryo test (SET) using *P. lividus* followed the tier I protocol described by Beiras et al.^16^ Plastic leachates were obtained according to the method described by Almeda et al.^34^ Compostable plastic samples were ground to 250 μm by mixing with dry ice in an Ultra Centrifugal Mill (Retsch ZM 200). For PE samples, which proved to be more difficult to micronize, a CryoMill (Retsch) was used to obtain particles of the desired size. Grinded samples were dried for 24 h at 20 °C in dark conditions. One g/L leachates were prepared in 65 mL glass bottles by shaking the micronized plastic in chemically defined artificial sea water (ASW)^35^ in an overhead rotator at 1 rpm for 24 h in dark conditions. The leachate was filtered through glass microfiber filters (Whatman, Grade GF/C 0.45 μm) and tested undiluted (×1) and in dilutions of ×1/3, ×1/10, and ×1/30 in filtered ASW (*n* = 4 per dilution). As a control, filtered ASW was used (*n* = 8). Physicochemical parameters were measured from both control and leachate samples to ensure that there were no changes in the medium properties. Sea urchins were provided by the ECIMAT stock, originally collected from natural habitats in the Ria de Vigo (NW Iberian Peninsula). Fertilized sea urchin eggs (40 mL^–1^) were incubated for 48 h in dark conditions at 20 ± 1 °C in 4 mL glass vials and, afterward, fixed with 6 drops per vial of 36% formaldehyde. Recently, González et al.^36^ described increased toxic effects of 2-phenylbenzimidazole-5-sulfonic acid (PBSA) when incubating *P. lividus* under light conditions. Therefore, additional incubations featuring light in the visible range were performed using BIO1 and PE specimens from the UV_T and UV_D treatments. Size recordings were done with Leica image analysis software LAS V4.12 and a Leica DMI 4000 B microscope with a 2.5× objective for larvae and a 5.0× objective for the eggs. Size increase, calculated as mean (*n* = 35) maximum dimension minus mean egg size at *t* = 0, was used as the endpoint.^37^

### Statistical Analysis

All statistical analyses were performed using IBM Statistics SPSS v. 25.

Half-maximal effect concentrations (EC_50_) were calculated by fitting the data of the control-corrected length (Δ*L*_c_) vs dilution to a Probit dose–response model as previously described by Beiras et al.^38^ Toxic units (TUs) were calculated as the inverse of the EC_50_ multiplied by the concentration of plastic used for obtaining the leachate, in this case, 1 g L^–1^.

To identify the impacts of light and dark incubations and weathering on the toxicity of plastic leachates, a generalized linear model (GLM) based on a γ distribution with a log-link function was used. The GLM featured the TU as a dependent variable, UV dose as a covariate and incubation (categorical: light/dark), plastic type, precipitation (categorical: yes/no), and weathering (categorical: artificially weathered/environmentally weathered/new) as factors. The model was built to analyze all main effects, as well as all 2 factorial interactions and the interaction between plastic type–precipitation–UV dose. *R*^2^ was calculated for the GLM based on the residual deviance and the null deviance.^39^

## Results

### Absorbance of the Plastics

**Figure 2:**
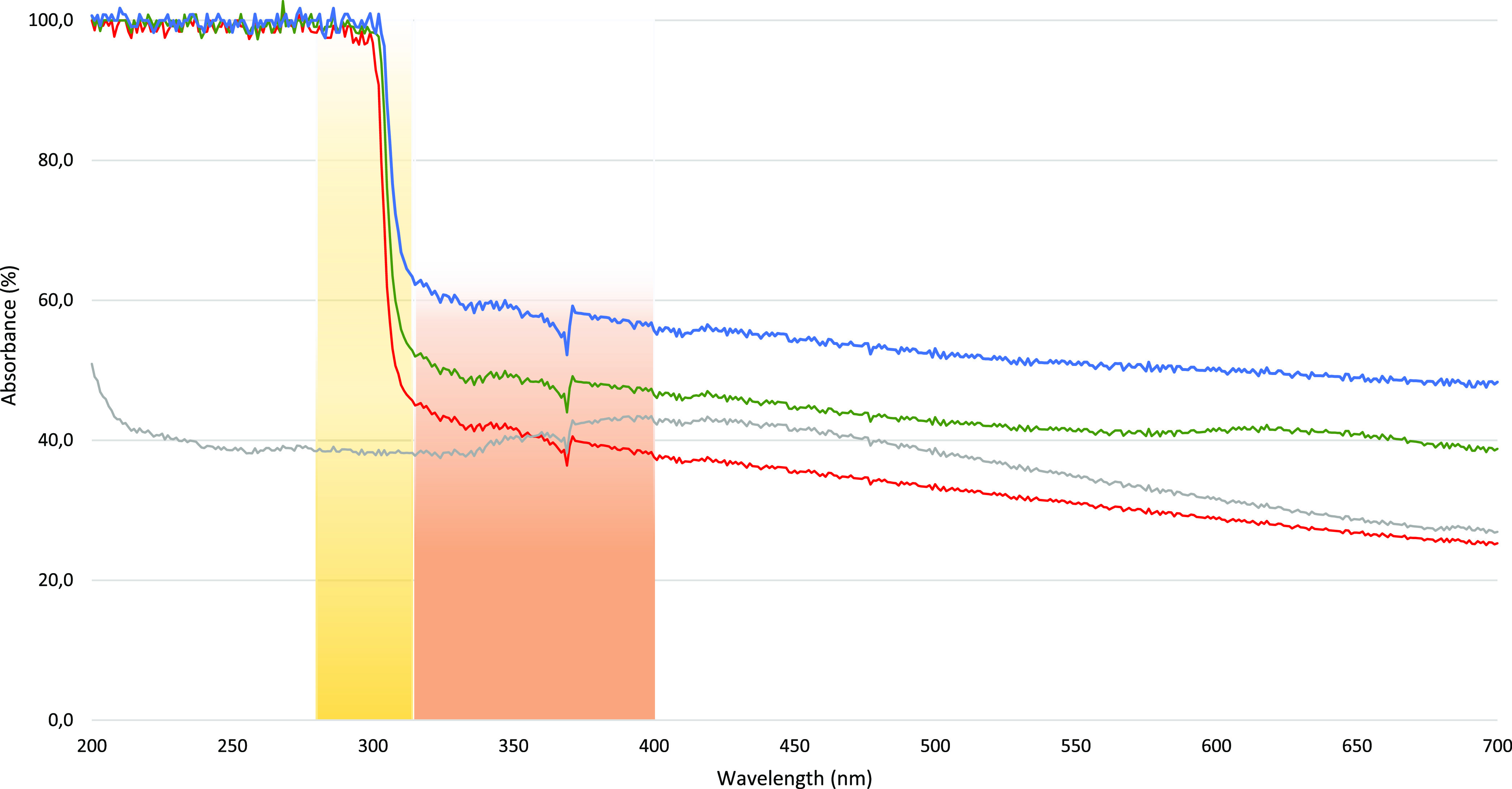
Absorbance of the four tested materials (BIO1—green, BIO2—blue, BIO3—red, and PE—gray) between 200 and 700 nm at *t*_0_. The UV-A and UV-B ranges are visualized by light orange and light yellow fading bands, respectively. Notice the high absorption shown by bioplastics but not by PE below 315 nm in the UV-B range.

Intact PE (*t*_0_) showed moderate absorbance throughout the UV-A (40.5 ± 2.0%), UV-B (38.4 ± 0.3%), and visible spectrum (VIS) (35.0 ± 5.3%). On the contrary, a much higher absorption, particularly in the UV-B spectrum, was measured for all three compostable materials (Figure 2). BIO2 showed the highest absorbance: 51.5 ± 2.4% in the visible spectrum, 58.4 ± 1.9% in the UV-A, and 91.5 ± 13.7% in the UV-B range. For BIO1, absorbances were 42.3 ± 2.1% (VIS), 48.6 ± 1.4% (UV-A), and 87.3 ± 18.3% (UV-B), and for BIO3, 31.1 ± 3.8% (VIS), 40.8 ± 2.0% (UV-A), and 82.9 ± 21.6% (UV-B) were measured. All three biomaterials showed the highest absorption in the UV-B spectral range, followed by the UV-A range. Interestingly, for these bioplastics, a sharp increase in absorbance absent for PE is observed below 315 nm (Figure 2).

### Temperature

#### Mesocosm Experiment

In the LIT_D exposure, temperatures did not exceed a maximum of 34.2 °C, with an overall mean temperature of 18.8 ± 4.9 °C and an average temperature range throughout the day of 10.0 ± 3.4 °C (Figure S1). The LIT and LIT_R treatment, on the other hand, experienced high temperature fluctuations with the LIT experiencing a maximum temperature on the sand surface of 60.9 °C, an average mean of 21.0 ± 10.0 °C, and a temperature range of 25 ± 8.5 °C throughout 24 h (Figure S2). In the LIT_R, similar temperatures were measured, with a maximum of 64.0 °C, an overall average of 24.9 ± 12.0 °C, and an average daily range of 31.4 ± 8.7 °C (Figure S3). An average air temperature during the test was 18.3 ± 3.5 °C, with a maximum temperature of 33.5 °C and an average temperature range of 5.7 ± 2.7 °C (Figure S4).

#### Artificial Weathering

Temperature within the test systems remained constant during the incubation time (Table 1). While the dark control maintained ambient temperature of around 22.5 ± 1.0 °C, the UV lamps increased the temperature in the UV_R system to 44.3 ± 2.3 °C and in the UV_T to 50.7 ± 1.0 °C. Overall, the spectra obtained showed high intensities between 310–316, 360–372, 400–410, and 430–441 nm, while environmental radiation presents more homogeneous intensities (Figure S5).

### UV Dose

In both experiments, the filtration of the UV light using a UV filtering foil successfully reduced the UV transmittance throughout the whole experiment with a transmittance of <1% for wavelengths below 370 nm (Figure S6).

The highest overall UV dose was reached in the Mesocosm-2021 exposure (*D*_UV28_ = 2,345,734 mJ cm^–2^; *D*_UV120_ = 11,378,583 mJ cm^–2^), followed by the Mesocosm-2022 exposure (*D*_UV28_ = 1,964,455 mJ cm^–2^; *D*_UV120_ = 10,963,192 mJ cm^–2^). Malfunctions were observed for the UV recording for 18 days in April. On these days, no data was acquired. To estimate the *D*_UV_ of April, we calculated the average *D*_UV_ per day based on the 12 measured days of April and added this value for each missing day. The artificial weathering experiment accounted for a *D*_UV_ = 2,584,989 mJ cm^–2^ for UV_T and *D*_UV_ = 75,494 mJ cm^–2^ for UV_R after 21 days (Table 1). In the natural exposures, UV-A accounted for the main radiation, while in the artificial weathering, the UV-B fraction was substantially higher. Even compared to the outside exposures, *D*_UV-B_ measured in UV_T was 26 times higher than the total UV-B dose measured previously^18^ (Table 1). The total simulated time in UV_T accounted for *t*_a_ = 28 days, and that in UV_R accounted for *t*_a_ = 1 day.

### Toxicity

While the new PE plastic was not toxic for sea-urchin embryos (<1 TU), all compostable plastics did provoke adverse effects (Table 2). In particular, BIO2 showed the highest toxicity with 3 TU, followed by BIO1 with 2.65 TU. Exposing the materials to environmental and artificial UV radiation resulted in no toxicity for PE in any of the treatments. Also, BIO1 showed no toxic effects on *P. lividus* embryos after 28 days of environmental exposure and BIO2 only showed slight toxicity in LIT_R (1.80 TU) and LIT_D (1.12 TU) but no toxic effects in LIT. For BIO2, toxicity disappeared after 120 days of exposure in all three treatments. On the contrary, the home-compostable bag BIO1 showed moderate toxicity (2.50 TU) again after 120 days of environmental exposure.

**Table 2:**
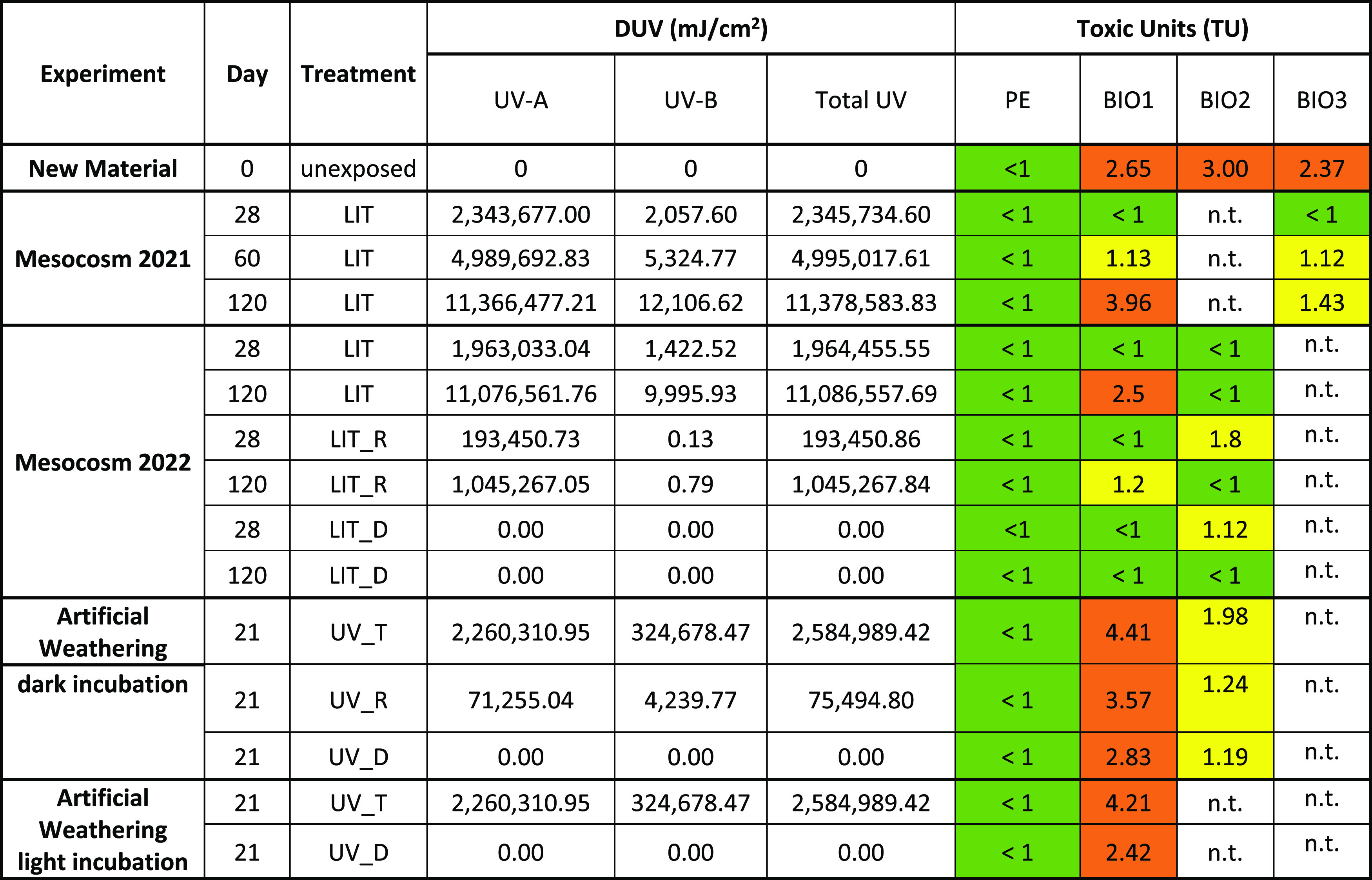
Summarized Results of the Sea-Urchin Embryo Test (SET) in Relation to UV Dosage (DUV)^a^

a Results are grouped by experiment and treatment. n.t. stands for not tested. Green corresponds to no toxicity; yellow, to slight toxicity, and orange, to relevant toxicity.

**Figure 3:**
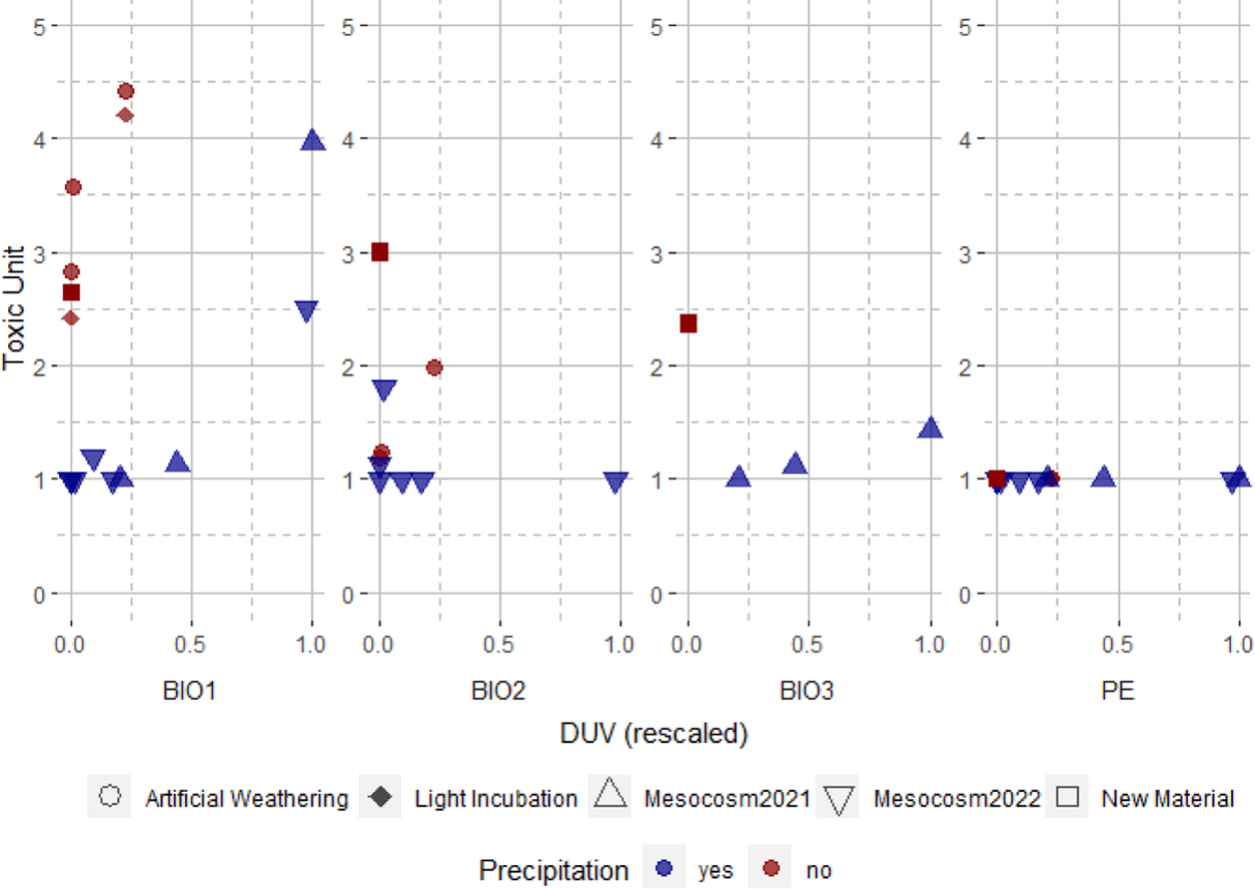
TU is shown in relation to the UV radiation dose (DUV), expressed as a proportion of the maximum dose. The UV dose is rescaled between 0 and 1 for easier viewing (DUV rescaled). Circles represent results from the artificial weathering experiment, diamonds represent the results from the sea-urchin embryo test conducted in light conditions, up facing triangle the Mesocosm-2021, downfacing triangles the Mesocosm-2022 and squares show the TU of the unexposed materials. The color code indicates if precipitation was present (blue) or absent (red) in the experiment.

The artificial weathering remarkably increased the toxicity of BIO1 to 4.41 TU in UV_T, and the strongest toxic effect was observed. Interestingly, for this material, toxicity was not lost in the UV_R treatment but increased to 3.57 TU. In UV_D, a 2.83 TU was calculated, similar to the new material’s initial toxicity. For BIO2, a similar pattern was found, even though the toxic effect was lower, showing its highest toxicity in UV_T treatment (1.98 TU) and followed by UV_R and UV_D with TUs of 1.24 and 1.19, respectively (Figure 3). Incubating sea urchin larvae under light did not lead to significant changes of the toxicity for BIO1 (UV_T: 4.21 TU, UV_D: 2.42 TU) or PE (UV_T: <1 TU, UV_D: <1 TU) (Figures 3, S9, and S10 and Table 2).

The GLM (*R*^2^ = 0.97) revealed significant effects of plastic type (*p* < 0.0001), UV dose (*p* < 0.0001), weathering (*p* < 0.0001), and precipitation (*p* < 0.0001) on the toxicity (Table TS2 and Figures S8 and S9). The factor incubation (light vs dark) during toxicity testing did not show significant effects (*p* = 0.11). Therefore, precipitation remarkably reduced the toxicity of weathered bioplastics, while the UV dose increased the bioplastics TU (Table TS4).

## Discussion

### Compostable vs Conventional Bags

Confirming previous findings,^18^ new commercial compostable bags consistently showed a remarkably higher toxicity (from 2.4 to 3.0 TU) compared to PE bags (<1 TU). Several bioplastics, including PBAT- and PLA-based materials, previously showed in vitro toxicity at both baseline and endocrine disruption levels.^7^ Moreover, in the present study, PE bags did not show any adverse effects on sensitive sea-urchin larvae disregarding light treatment (see Table 2). This may be due to the low-to-medium photoactivity of PE, here confirmed by its low absorbance in the UV spectra compared to bioplastic bags (see Figure 2), making it less suitable to undergo UV-induced transformations or photodegradation.^40^ As shown in Figure 2, PE showed low visible and especially UV light absorbance, in line with previous findings.^23^ In contrast, the strong absorption of the bioplastics in the UV range is likely the result of electronic transitions of chromophoric groups,^41^ necessary for light absorption and thus for the activation of photochemical reactions.^42^ Chromophores can be, among others, double bonds and aromatic rings, structures contained in the tested bioplastics (Table TS1). But as solid polymer materials are composed of numerous absorbent systems in the polymer chain, as well as several UV absorbing additives, such as antioxidants, light stabilizers, and plasticizers, only broad, unspecific bands are visible in the absorbance spectra (Figure 2), making the identification of the exact polymer structure and additives impossible.^41^ Nonetheless, the higher UV absorption of the tested bioplastics most likely results in more photodegradation^43^ and therefore could facilitate the leaching of hazardous substances^20,44^ or the formation of toxic transformation products.^22,27,45,46^

### Effects of Weathering on Toxicity

In our previous study using underwater weathering conditions,^18^ we hypothesized that the rapid loss of toxicity observed during the first days of weathering was due to mobilization of unbound additives from the polymeric matrix into the surrounding water. In the present study, we found that exposure to rain (see Figure S11) and humidity plays a similar role in the development of toxicity during weathering, and after 28 days of outdoor exposure, the initial toxicity of the brand-new materials (2.4–3.0 TU) virtually disappeared (from <1 to 1.8 TU). Chemical additives have been proved to be the main cause of plastic toxicity,^11,47^ and their rapid leaching has been described before.^15,48^ In order to avoid this, an indoor weathering experiment in dry conditions was conducted. Under these conditions, home-compostable bags tested did not show any reduction in toxicity (2.4–3.6 TU after 21 days in dark and UV-screened light), while the industrial compostable bag showed a reduction in toxicity (1.19 TU). When irradiating the bags with UV light of different intensities, toxicity increased in both samples compared to the dark control, indicating that even moderate dosages of UV can facilitate adverse effects of bioplastic materials (see Figure 3 and Table 2). Higher total UV light dosages were achieved after long exposure times (up to 120 days) in outdoor conditions. Despite the likely initial leaching of additives, those high UV doses counterbalanced the leaching effect in the case of the BIO1 bag, and eventually, the toxicity of outdoors weathered samples could be modeled as a function of the total UV dose received, in mJ cm^–2^, according to the expression
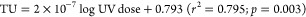
BIO3, used in the 2021 mesocosm only, showed a much smaller UV-dose-dependent induction of toxicity, whereas BIO2, used in 2022, did not show the same trend. Therefore, quantitative modeling of the impact of UV dose on toxicity using the pooled data from all compostable bags could not be attempted. We must bear in mind that only BIO1 bags were certified as home compostable, whereas BIO2 and BIO3 were compostable in industrial facilities only.

### Influence of Temperature

In the mesocosm exposures, high temperatures were experienced on the surface of the LIT and LIT_R treatments. Given the relatively low specific heat capacity of sand,^49^ high surface temperatures are commonly seen on sandy grounds in the open environment as well.^50^ In artificial weathering experiments, toxicity increased with higher UV dose and temperature, following the order UV_T > UV_R > UV_D. However, in the mesocosm exposure, toxicity, temperature and UV light did not covariate: a higher temperature was reached in LIT_R, followed by LIT, but toxicity remained higher in LIT, the treatment receiving the higher UV dose. Because of this, we conclude that temperature does not play a major role in the toxicity development of the tested materials. On top, the possible influence of wind and precipitation was successfully removed in the artificial weathering. Unfortunately, direct rain was also removed from the LIT_R and LIT_D setup, but visible and tactile evaluations showed high moisture in the sand and condensational water at the lids and underneath the samples, predominantly in the mornings. Lastly, the artificial weathering and environmental exposure were able to reduce the UV radiation to 1% in LIT_R and UV_R or completely block off direct light in LIT_D and remove all light in UV_D.

### Influence of UV Dose

The experimental results of this study unveil UV dose as a major driver of bioplastic toxicity evolution under both outdoor natural conditions and indoor artificial light exposures, in sharp contrast with the lack of response of the conventional PE materials. A greater UV dose led to stronger toxic effects for the home-compostable material BIO1 and the industrial compostable material BIO3 under all tested conditions and for the industrial compostable BIO2 material under artificial light exposure but not in the mesocosm experiment. BIO2 was tested in mesocosm a different year than BIO3, and natural weather differences may be responsible for these contrasting results.

This study confirms that the initial toxicity of compostable bags is rapidly lost after environmental exposure,^31^ in line with rapid leaching of additives, not covalently bond to polymeric chains, in aquatic environments.^13^ Here, we found that in the home-compostable material and to some extent in the industrial compostable materials, this increase in toxicity throughout weathering is chiefly dependent on the UV dose, most likely due to the formation of degradation products.^22^ As further discussed in the next section, UV-driven photo-oxidation of organic molecules produces hydroxylated derivatives more bioavailable and toxic than parental compounds.^51^

An additional mechanism that can contribute to explain the toxicity increase is the higher availability of toxic substances due to weathering of the material since the affinity of the polymeric matrix for organic chemicals can be strongly affected by the chemical changes caused by weathering processes.^52^ In fact, it has been experimentally demonstrated that plastic materials continue to leach substances that negatively affect aquatic organisms for periods beyond 100 days.^53^ Furthermore, synergistic effects of the initial toxic additives and the newly formed toxic products could play a role in artificial weathering trials. Synergistic effects of microplastics and functional additives have been described before for freshwater organisms,^54^ but knowledge targeting marine life is scarce. Given the broad number of plastic additives and possible transformation products,^22,55^ tracking the exact reason for the toxic effects remains hard.

The risk weathered plastic poses is widely discussed,^46,56^ and the toxic effect of weathered conventional and bioplastic is well known,^27,28,57^ even exceeding the effect found in new materials.^45^ Here, we demonstrate that the dose of UV received by a material is crucial for its toxicological evaluation, including risk assessment studies, and we propose to integrate this knowledge in future standards and regulations.

## Environmental Implications

Land-based plastic is a major contributor to the pollution of oceans.^58−60^ Once in the marine environment, plastic can stay afloat or sink^61^ and is subject to wind, currents, and tides, leading to beaching of plastic in shorelines.^62^ Compostable plastics were shown to only moderately degrade in marine environments,^63,64^ especially in pelagic habitats,^18,65,66^ and thus are likely to find their way into littoral habitats again, as easily observed in the upper intertidal zone of shoreline, where they cause ecological impacts.^8,9,67^ Plastic fragmentation processes are enhanced under the high temperature and irradiance conditions prevailing in many coastal areas, leading to smaller particles^68^ posing even higher risk to organisms.^69,70^ Our findings support that environmentally weathered bioplastics can pose a risk to marine and coastal organisms even when plastic additives are rapidly leached and initial toxicity is temporarily lost. Since the present experiments were conducted under controlled conditions (filtered oceanic seawater, artificial sand), adverse effects can be provoked not only by chemicals potentially resorbed from the environment^46^ but also by intrinsic properties of the degraded materials themselves.

Similar to other studies of this kind, concentrations used for the toxicological assessment are several orders of magnitude above environmental concentrations of plastic particles reported in the oceans.^70^ Nonetheless, particle densities vary highly depending on marine habitat,^62^ with coastline and beaches receiving a considerably high amount of weathered plastics.^60,71^ High temperatures,^72^ UV irradiances,^73,74^ and hydrodynamics^75^ maximize the fragmentation of plastic objects and the formation of secondary microplastics in the shores.^76,77^ Under these conditions, the patterns of toxicity change associated with weathering here described may acquire environmental relevance.

The increase of toxicity undergone by organic aromatic molecules when exposed to UV light is well known.^51^ Some polycyclic aromatic hydrocarbons (PAHs) are photoactivated upon light exposure and their toxicity to early life stages of marine invertebrates increases.^78^ The matrix of all compostable bags was identified by FTIR as a terephthalate (aromatic) polyester, and photoactivation of the phthalic radical is expected upon strong UV exposure. However, the present study did not attempt the chemical analyses of weathered functional groups or metabolites, and thus, the driver for increased toxic effects remains unknown. Future studies could target the degradation products of those aromatic radicals to provide further insights into the modes of action of weathered bioplastics.

Mesocosm experiments were conducted from spring to summer, with decreasing precipitation toward summer (Figure S11). Due to the lack of rain in the later stages of the experiment, it remains unclear if gained toxic properties could be easily lost by leaching of metabolites into rainwater. Still, as experiments in 2 consecutive years showed similar results, we expect the outcome to be representative at least for temperate coastal areas. Regarding artificial weathering, the light sources were chosen to maximize UV radiation dose and thus shorten experiment length and reduce costs. Consequently, a substantially higher amount of UV-B radiation compared to environmental conditions was observed. More environmentally, realistic approaches can be obtained by using xenon lamps that produce light spectra more similar to sunlight.

## Supporting Information Available

It contains detailed information about the plastic materials used in this study, environmental conditions in the mesocosm test system, measured UV spectra in the artificial and environmental weathering experiment, verification spectra of the UV filtration units, as well as more detailed results of the statistical analyses. Furthermore, original and additional data is available under Summary_UVimpacttoxicity.xlsx, containing results of the toxicological tests, EC_50_ concentrations, as well as the raw data used for graphs in this article and Supporting Information (PDF)

## Abbreviations

ASW: artificial sea water
BBP: bio-based plastics
BDCP: biodegradable and compostable plastics
BIO1: bioplastic 1
BIO2: bioplastic 2
BIO3: bioplastic 3
*D*_UV_: UV dose
*D̅*_UV_: UV dose per day
*D*_UV art_: artificial UV dose
*D*_UV env_: environmental UV dose
GLM: generalized linear model
LIT: littoral treatment
LIT_D: littoral shaded treatment
LIT_R: littoral reduced UV treatment
LR: likelihood ratio Chi square
PAH: polycyclic aromatic hydrocarbons
PBAT: polybutylene adipate terephthalate
PBSA: 2-phenylbenzimidazole-5-sulfonic acid
PE: polyethylene
PHA: polyhydroxyalkanoate
PLA: polylactide
PVC: poly(vinyl chloride)
SET: sea-urchin embryo test
*t*_a_: artificially weathered days
TU: toxic unit
UV: ultraviolet
UV_D: artificial weathering dark exposure
UV_R: artificial weathering reduced UV exposure
UV_T: artificial weathering full UV exposure
Δ*L*_c_: control-corrected length
χ^2^: Chi square
