# Supplementary material for: UV Dosage Unveils Toxic Properties of Weathered Commercial Bioplastic Bags
Source: Environ Sci Technol. 2023 Sep 26;57(40):14807–16. doi: 10.1021/acs.est.3c02193 (PMC10569051; doi:10.1021/acs.est.3c02193)
Supplement: Supplementary file 1 — es3c02193_si_001.pdf [file es3c02193_si_001.pdf]

Supporting Information

UV dosage unveils toxic properties of weathered commercial bioplastic bags

Jakob Quade[1], Sara López-Ibáñez[1], Ricardo Beiras[1,2].*

[1] ECIMAT-CIM, *Universidade de Vigo*. Illa de Toralla, 36331 Vigo, Galicia, Spain

[2] Facultade de Ciencias do Mar, Universidade de Vigo, 36310, Vigo, Galicia, Spain

[*]Corresponding author: salopez@uvigo.es

Summary - 12 pages, 4 tables, 12 graphs

Name (Internal Sample Code)	BIO1	BIO2	BIO3	PE
Picture	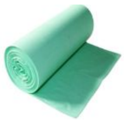	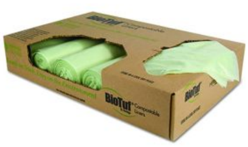	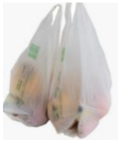	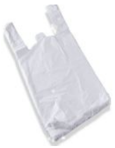
Manufacturer description	100% biodegradable bag made from corn starch	Compostable in industrial facilities which may not be available in all areas. Not suitable for backyard composting.	Biodegradable and Compostable T-shirt bag with green print	T-shirt bag LDPE
Brand	Green Maker	Heritage	Eco Pack	Pampols
Access Online	https://www.amazon.es/dp/B07FSBYHD8/ref=twister_B07KP9RCFH?_encoding=UTF8&th=1	https://www.kleensupply.com/catalog/p/HER+Y7658TER01/Heritage-Biotuf-Compostable-Can-Liners-60-gal-09-mil-38-x-58-Green-20-Bags-Roll-5-Rolls-Carton/	https://envasesecopack.com/products/bolsas-compostables-con-asa-small-200-2x60x400mm	https://www.amazon.es/BOLSAS-CAMISETA-35X50-Blanca-unidades/dp/B079Q487JS?th=1
Composition according to Manufacturer	PLA/PBAT + Maize starch	Bioplast (PLA)	Maize starch + unidentified compounds	LDPE
FTIR analysis	Polyester-terephthalate	Polyester-terephthalate + other esters + talcum	Polyester-terephthalate	Polyethylene
Certification/ Labelling	Certified EN-13432 and “home” compostable by TÜV Austria, and with standard Seedling logo	BPI Certified Compostable. Conforms to ASTM D6400 Standard	Certified EN-13432 and “industrial” compostable by TÜV Austria	-
Fabrication area	China	USA	Spain	Spain
Colour	Green	Light green	Translucent Beige	White
Type and volume	6-L sack	50-L sack	6-L carrier bag	10-L carrier bag
Thickness (µm)	18	22	20	15

Table TS1 Characteristics of the tested materials. FTIR analysis was conducted by CACTI (University of Vigo).

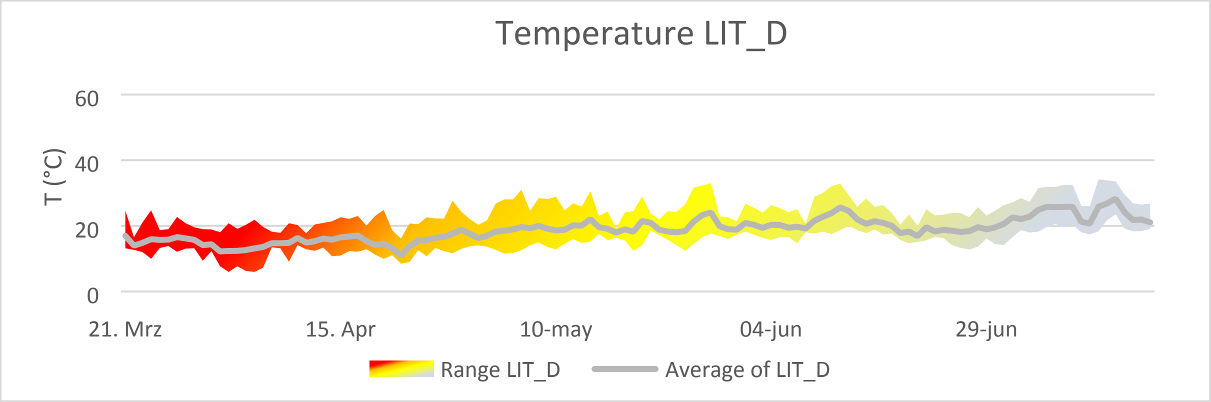

Figure S1 Temperature recorded on top of the sand in the littoral shaded exposure (LIT_D) throughout the experimental period. The range is calculated as the result of maximum and minimum temperature throughout 24h.

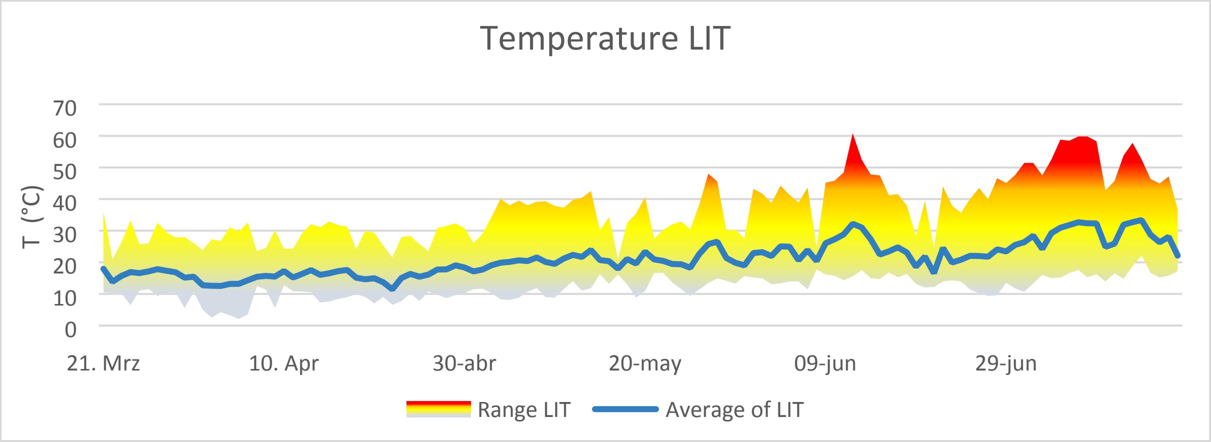

Figure S2 Temperature recorded on top of the sand in the littoral natural exposure (LIT).

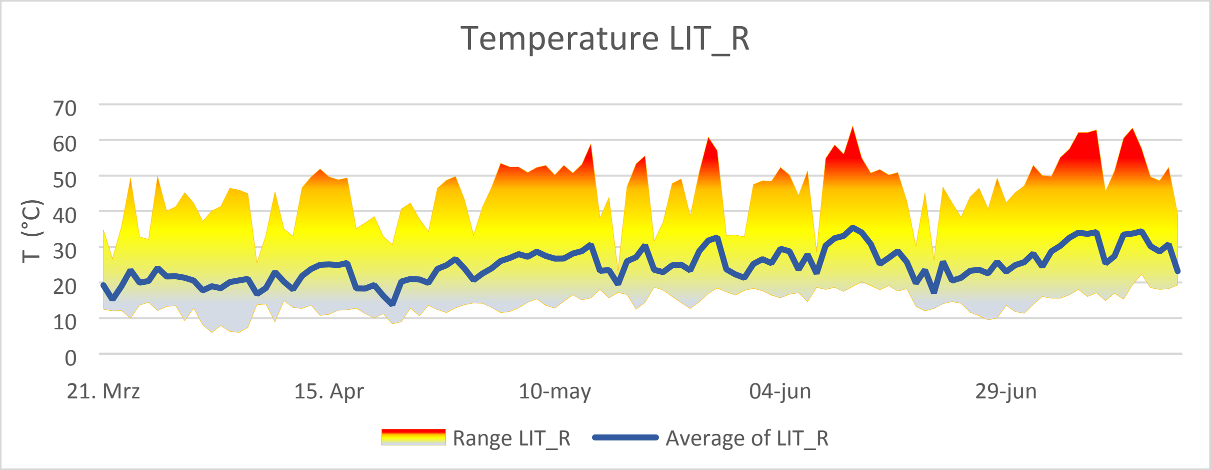

Figure S3 Temperature recorded on top of the sand in the littoral exposure with reduced UV irradiation (LIT_R). Notice that the UV screen caused a minor increase in temperature.

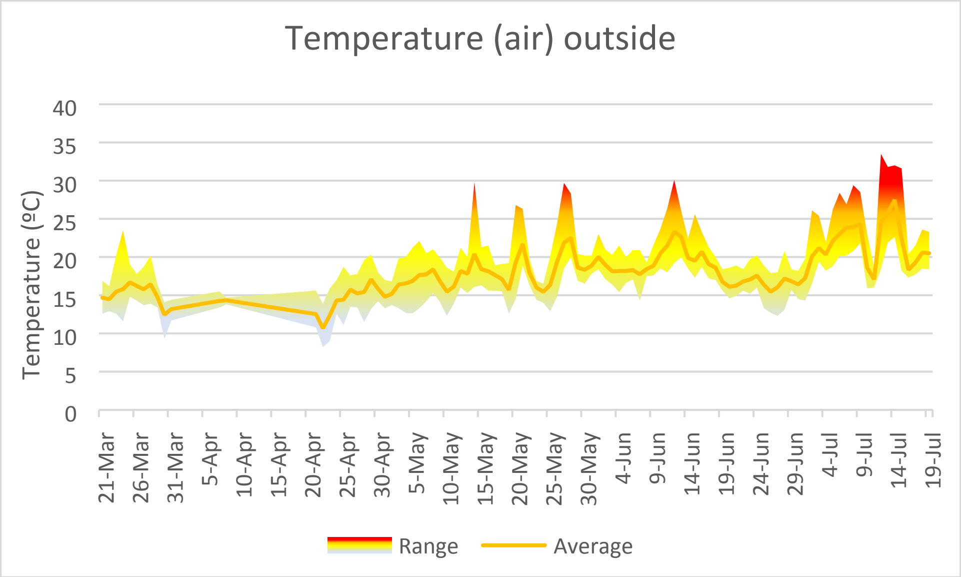

Figure S4 Air temperature recorded in the mesocosm facility during the experimental period.

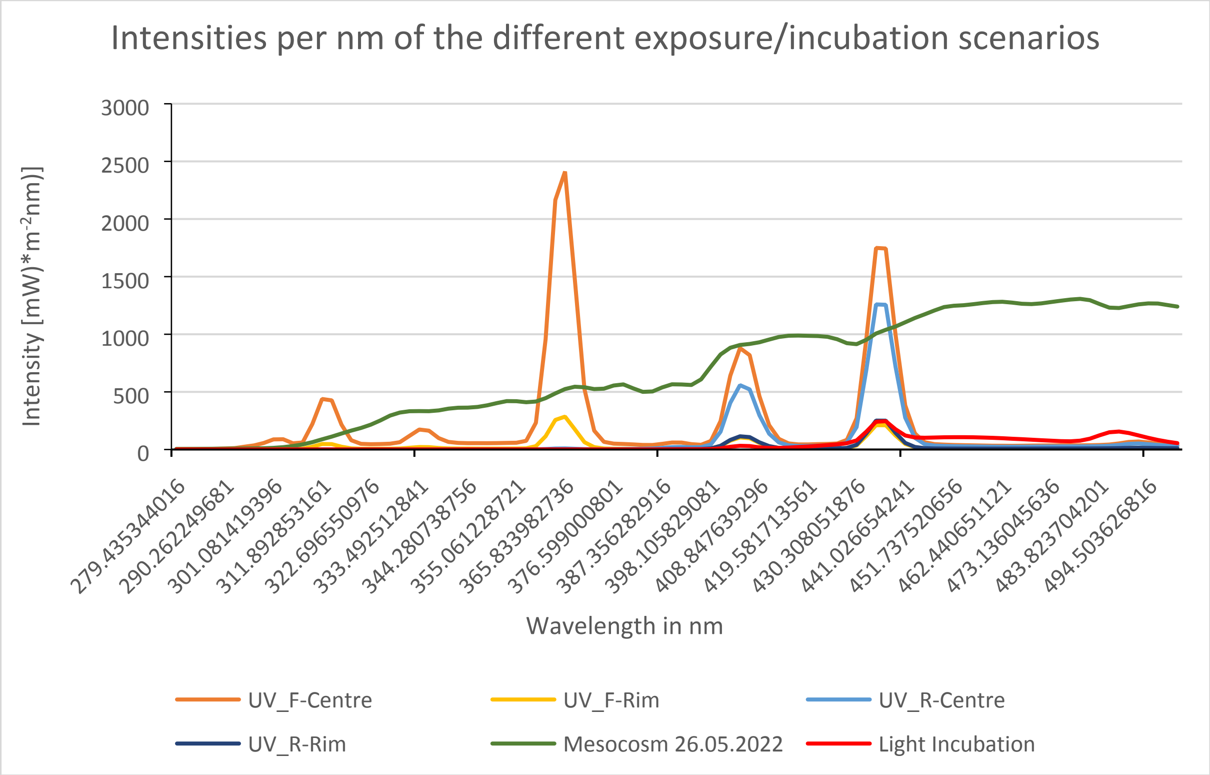

Figure S5 Light spectra recorded in the mesocosms and in the artificial exposures. The mesocosm spectrum (green) was taken at mid-day in a day with average weather conditions. For the artificial weathering experiment (UV_F -(orange, yellow); UV_R (light blue, dark blue)) measurements were taken in the centre and the rim. The light incubation (red) measurement shows the spectrum of the light used for the light incubation experiment.

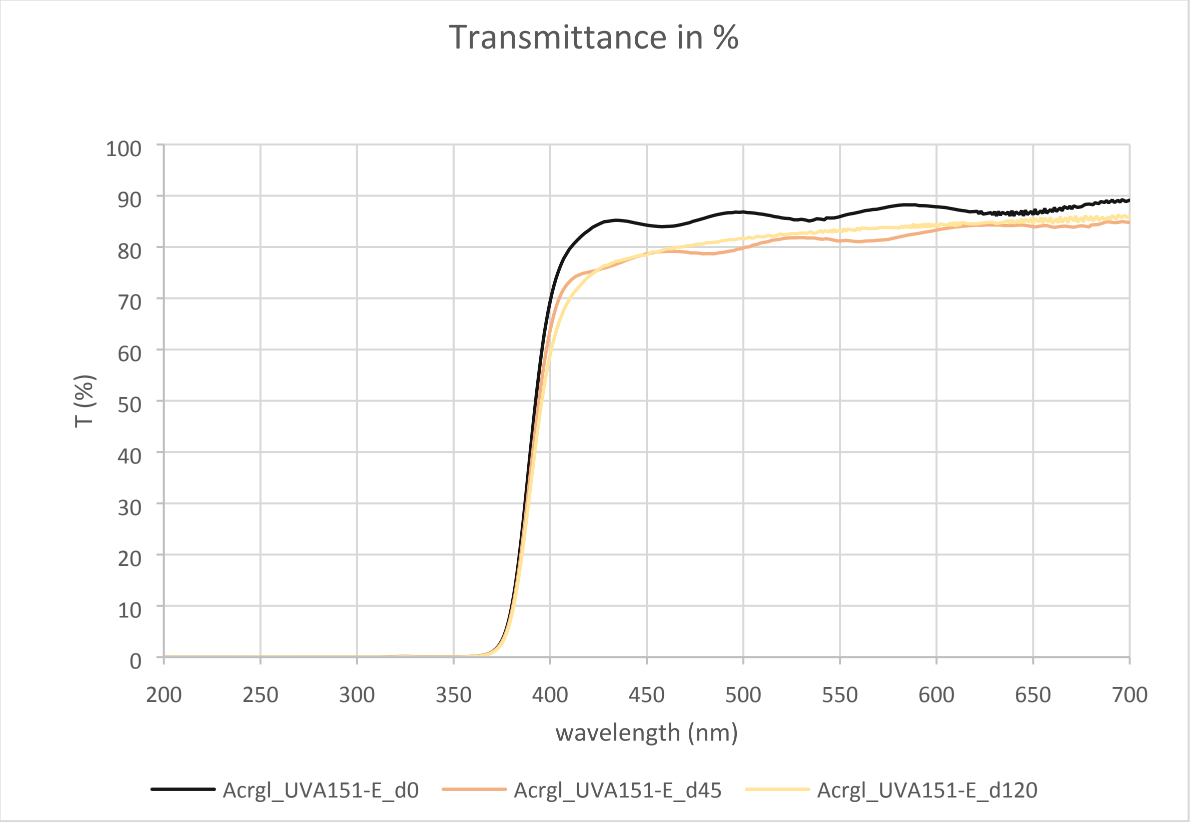

Figure S6 Transmittance (T) of the UV filtration unit (acrylic glass+ UV reducing film) used in the mesocosm and artificial weathering experiments. Acrgl_UVA151-E_d0 (black) presents the data for day 0, Acrgl_UVA151-E_d45 (orange) was measured at day 45 of the mesocosm experiment and Acrgl_UVA151-E_d120 (yellow) was measured after the experiment took place. Notice the effective reduction of radiation below 380 nm.

Tests of Model Effects

Source	Type III Likelihood Ratio Chi-Square	df	Sig.
(Intercept)	68.734	1	.000
Plastic Type	78.645	3	.000
Precipitation	53.534	1	.000
weathering	23.040	1	.000
Incubation	2.622	1	.105
UV-dose	19.924	1	.000
Plastic Type * Precipitation	81.932	2	.000
Plastic Type * weathering	61.960	2	.000
Plastic Type * Incubation	2.658	1	.103
Plastic Type * UV-dose	17.323	3	.001
Precipitation * weathering	.[a]	.	.
Precipitation * Incubation	.[a]	.	.
Precipitation * UV-dose	8.173	1	.004

weathering * Incubation	.[a]	.	.
weathering * UV-dose	.[a]	.	.
Incubation * UV-dose	.769	1	.380
Plastic Type * Precipitation * UV-dose	11.493	2	.003
Plastic Type * Incubation * UV-dose	.771	1	.380

Dependent Variable: Toxic Unit

Model: (Intercept), Plastic Type, Precipitation, weathering, Incubation, UV-dose, Plastic Type * Precipitation, Plastic Type * weathering, Plastic Type * Incubation, Plastic Type * UV-dose, Precipitation * weathering, Precipitation * Incubation, Precipitation * UV-dose, weathering * Incubation, weathering * UV-dose, Incubation * UV-dose, Plastic Type * Precipitation * UV-dose, Plastic Type * Incubation * UV-dose

a. Unable to compute because the estimable function has zero degrees of freedom.

*Table TS2 Results of the GLM performed with BIO1, BIO2, BIO3 and PE, including toxic units as dependent variable, UV dosage (DUV) as covariate and precipitation, plastic type (Plstc), status (weathered/new) and incubation (light/dark) as factors and all possible interactions. Significance (Sig.) was accepted if p<0.05. Tested interactions are marked with an asterisk * in row 1. Partial eta squared describes the effect size, df represents the degrees of freedom and F the F-value. The first row describes the model parameters and settings.*

Goodness of Fit[a]

	Value	df	Value/df
Deviance	.584	43	.014
Scaled Deviance	43.000	43	
Pearson Chi-Square	.632	43	.015
Scaled Pearson Chi-Square	46.527	43	
Log Likelihood[b,c]	-92.868		
Adjusted Log Likelihood[d]	28.591		
Akaike's Information Criterion (AIC)	227.736		
Finite Sample Corrected AIC (AICC)	249.736		
Bayesian Information Criterion (BIC)	273.072		
Consistent AIC (CAIC)	294.072		

Dependent Variable: Toxic Unit

Model: (Intercept), Plastic Type, Precipitation, weathering, Incubation, UV-dose, Plastic Type * Precipitation, Plastic Type * weathering, Plastic Type * Incubation, Plastic Type * UV-dose, Precipitation * weathering, Precipitation * Incubation, Precipitation * UV-dose, weathering * Incubation, weathering * UV-dose, Incubation * UV-dose, Plastic Type * Precipitation * UV-dose

a. Information criteria are in smaller-is-better form.

b. The full log likelihood function is displayed and used in computing information criteria.

c. The log likelihood is based on a scale parameter fixed at 1.

d. The adjusted log likelihood is based on an estimated scale parameter and is used in the model fitting omnibus test.

Table TS3 Information about the goodness of the used model. A low deviance thereby represents a higher model fit.

Parameter Estimates

			95% Profile Likelihood Confidence Interval		Hypothesis Test		
Parameter	B	Std. Error	Lower	Upper	Wald Chi-Square	df	Sig.
(Intercept)	-.048	.1362	-.312	.222	.122	1	.727
[Plastic Type=BIO1]	-.256	.1685	-.587	.074	2.312	1	.128
[Plastic Type=BIO2]	.186	.0753	.039	.334	6.109	1	.013
[Plastic Type=BIO3]	-.089	.1394	-.357	.189	.403	1	.525
[Plastic Type=PE]	0[a]	.	.	.	.	.	.
[Precipitation=no]	.017	.0957	-.169	.207	.031	1	.861
[Precipitation=yes]	0[a]	.	.	.	.	.	.
[weathering=N]	-.017	.0939	-.203	.165	.032	1	.859
[weathering=W]	0[a]	.	.	.	.	.	.
[Incubation=Dark]	.048	.1263	-.203	.293	.142	1	.706
[Incubation=Light]	0[a]	.	.	.	.	.	.
UV-dose	4.287E-8	6.0762E-8	-7.664E-8	1.617E-7	.498	1	.480
[Plastic Type=BIO1] * [Precipitation=no]	1.203	.1303	.947	1.458	85.144	1	.000
[Plastic Type=BIO1] * [Precipitation=yes]	0[a]	.	.	.	.	.	.
[Plastic Type=BIO2] * [Precipitation=no]	-.016	.1387	-.287	.256	.013	1	.910
[Plastic Type=BIO2] * [Precipitation=yes]	0[a]	.	.	.	.	.	.

[Plastic Type=BIO3] * [Precipitation=no]	.951	.1548	.644	1.251	37.778	1	.000
[Plastic Type=BIO3] * [Precipitation=yes]	0[a]	.	.	.	.	.	.
[Plastic Type=PE] * [Precipitation=no]	0[a]	.	.	.	.	.	.
[Plastic Type=PE] * [Precipitation=yes]	0[a]	.	.	.	.	.	.
[Plastic Type=BIO1] * [weathering=N]	-.148	.1280	-.399	.103	1.341	1	.247
[Plastic Type=BIO1] * [weathering=W]	0[a]	.	.	.	.	.	.
[Plastic Type=BIO2] * [weathering=N]	.928	.1345	.664	1.192	47.625	1	.000
[Plastic Type=BIO2] * [weathering=W]	0[a]	.	.	.	.	.	.
[Plastic Type=BIO3] * [weathering=N]	0[a]	.	.	.	.	.	.
[Plastic Type=BIO3] * [weathering=W]	0[a]	.	.	.	.	.	.
[Plastic Type=PE] * [weathering=N]	0[a]	.	.	.	.	.	.
[Plastic Type=PE] * [weathering=W]	0[a]	.	.	.	.	.	.
[Plastic Type=BIO1] * [Incubation=Dark]	.176	.1525	-.123	.475	1.336	1	.248
[Plastic Type=BIO1] * [Incubation=Light]	0[a]	.	.	.	.	.	.
[Plastic Type=BIO2] * [Incubation=Dark]	0[a]	.	.	.	.	.	.
[Plastic Type=BIO3] * [Incubation=Dark]	0[a]	.	.	.	.	.	.
[Plastic Type=PE] * [Incubation=Dark]	0[a]	.	.	.	.	.	.
[Plastic Type=PE] * [Incubation=Light]	0[a]	.	.	.	.	.	.
[Plastic Type=BIO1] * UV-dose	1.047E-7	1.2538E-8	8.007E-8	1.292E-7	69.692	1	.000
[Plastic Type=BIO2] * UV-dose	-1.945E-8	1.4892E-8	-4.837E-8	1.006E-8	1.706	1	.192
[Plastic Type=BIO3] * UV-dose	3.935E-8	1.9932E-8	4.843E-10	7.869E-8	3.898	1	.048

[Plastic Type=PE] * UV-dose	0[a]	.	.	.	.	.	.
[Precipitation=no] * [weathering=N]	0[a]	.	.	.	.	.	.
[Precipitation=no] * [weathering=W]	0[a]	.	.	.	.	.	.
[Precipitation=yes] * [weathering=W]	0[a]	.	.	.	.	.	.
[Precipitation=no] * [Incubation=Dark]	0[a]	.	.	.	.	.	.
[Precipitation=no] * [Incubation=Light]	0[a]	.	.	.	.	.	.
[Precipitation=yes] * [Incubation=Dark]	0[a]	.	.	.	.	.	.
[Precipitation=no] * UV-dose	-1.859E-8	5.0115E-8	-1.159E-7	8.069E-8	.138	1	.711
[Precipitation=yes] * UV-dose	0[a]	.	.	.	.	.	.
[weathering=N] * [Incubation=Dark]	0[a]	.	.	.	.	.	.
[weathering=W] * [Incubation=Dark]	0[a]	.	.	.	.	.	.
[weathering=W] * [Incubation=Light]	0[a]	.	.	.	.	.	.
[weathering=N] * UV-dose	0[a]	.	.	.	.	.	.
[weathering=W] * UV-dose	0[a]	.	.	.	.	.	.
[Incubation=Dark] * UV-dose	-4.287E-8	6.0087E-8	-1.603E-7	7.533E-8	.509	1	.476
[Incubation=Light] * UV-dose	0[a]	.	.	.	.	.	.
[Plastic Type=BIO1] * [Precipitation=no] * UV-dose	6.097E-8	6.0903E-8	-5.844E-8	1.804E-7	1.002	1	.317
[Plastic Type=BIO1] * [Precipitation=yes] * UV-dose	0[a]	.	.	.	.	.	.
[Plastic Type=BIO2] * [Precipitation=no] * UV-dose	2.300E-7	7.6113E-8	8.123E-8	3.798E-7	9.135	1	.003

[Plastic Type=BIO2] * [Precipitation=yes] * UV-dose	0[a]	.	.	.	.	.	.
[Plastic Type=BIO3] * [Precipitation=no] * UV-dose	0[a]	.	.	.	.	.	.
[Plastic Type=BIO3] * [Precipitation=yes] * UV-dose	0[a]	.	.	.	.	.	.
[Plastic Type=PE] * [Precipitation=no] * UV-dose	0[a]	.	.	.	.	.	.
[Plastic Type=PE] * [Precipitation=yes] * UV-dose	0[a]	.	.	.	.	.	.
(Scale)	.014[b]						

Dependent Variable: Toxic Unit

Model: (Intercept). Plastic Type. Precipitation. weathering. Incubation. UV-dose. Plastic Type * Precipitation. Plastic Type * weathering. Plastic Type * Incubation. Plastic Type * UV-dose. Precipitation * weathering. Precipitation * Incubation. Precipitation * UV-dose. weathering * Incubation. weathering * UV-dose. Incubation * UV-dose. Plastic Type * Precipitation * UV-dose

a. Set to zero because this parameter is redundant.

b. Computed based on the deviance.

Table TS4 Estimated Coefficients are shown (B) and their significance are reported. A negative B indicates a negative impact, a positive B a positive impact of the parameter on the dependent variable toxic unit.

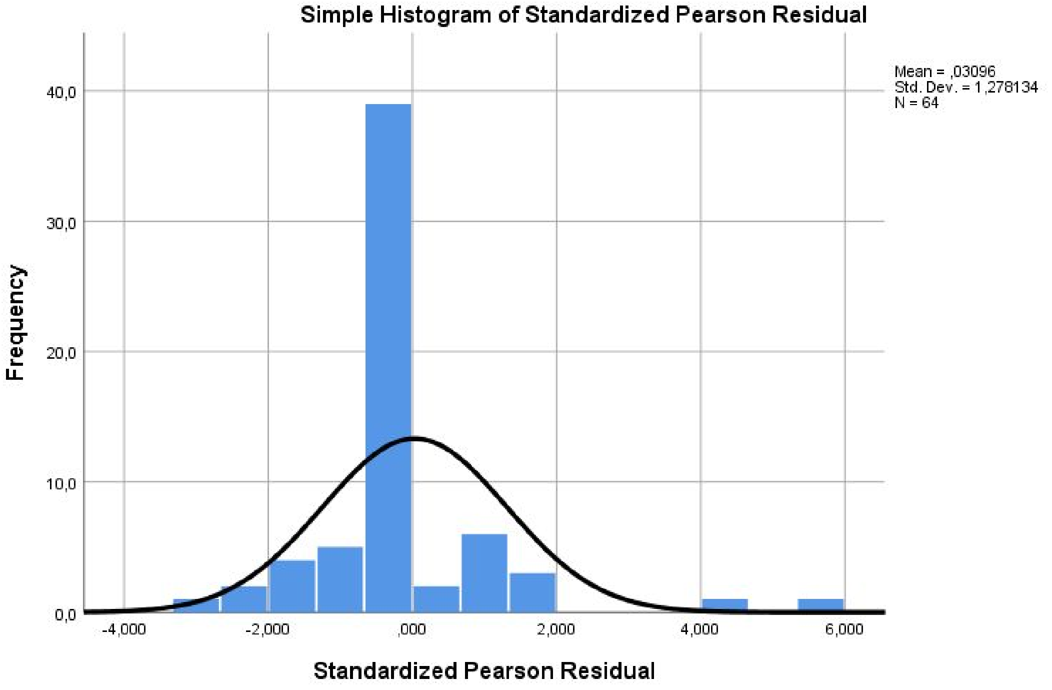

Figure S7 Distribution histogram of the standardized Pearson residuals for toxic units (TU) obtained with the GLM TS3. Based on this, we accept the normal distribution.

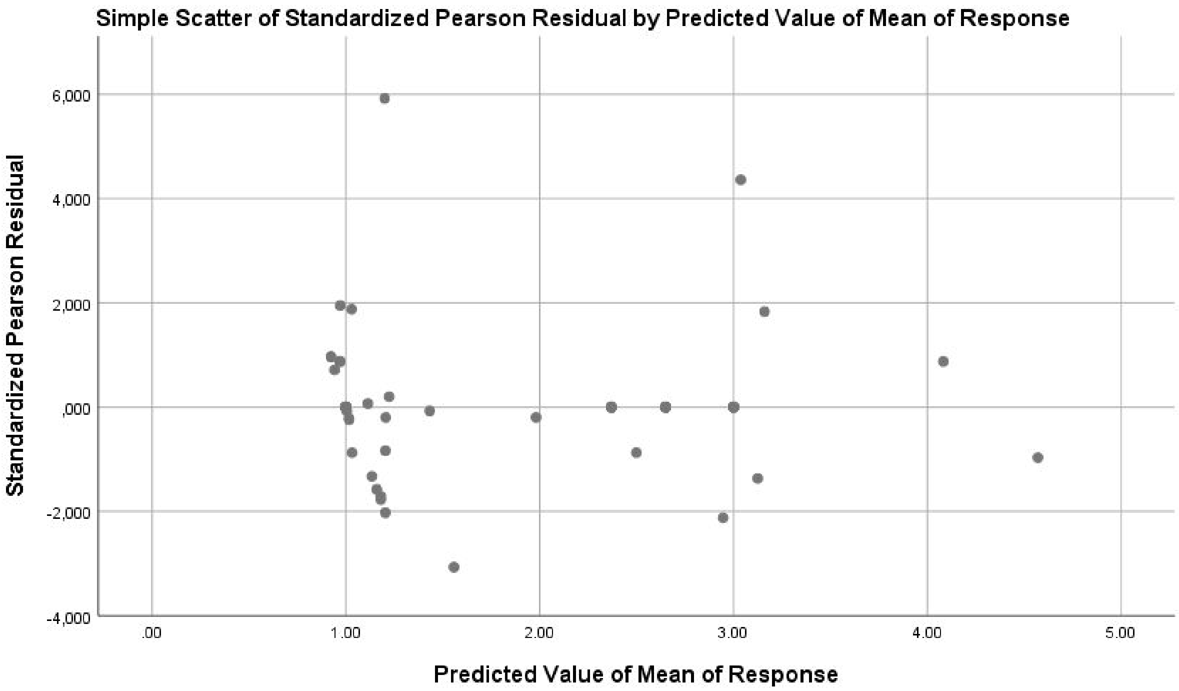

Figure S8 *Scatterplot of the predicted values and the standardized Pearson residuals obtained with the GLM TS3. No pattern is observed for the predicted values.*

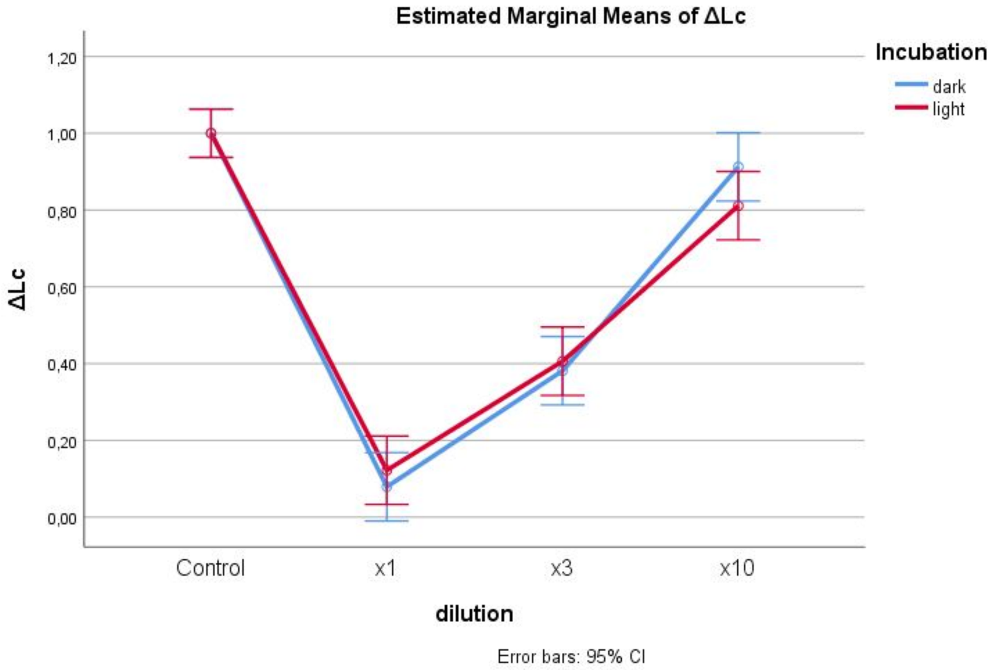

Figure S9 Comparison of light and dark incubation for BIO1 SET results. The figure shows the mean averages and 95% confidence intervals (CI) for sea-urchin larval length increase (ΔLc). The x-axis represents the 4 dilutions tested. Clearly overlapping CI´s indicate no significant difference in larval length between the light (pink) and dark (blue) incubation.

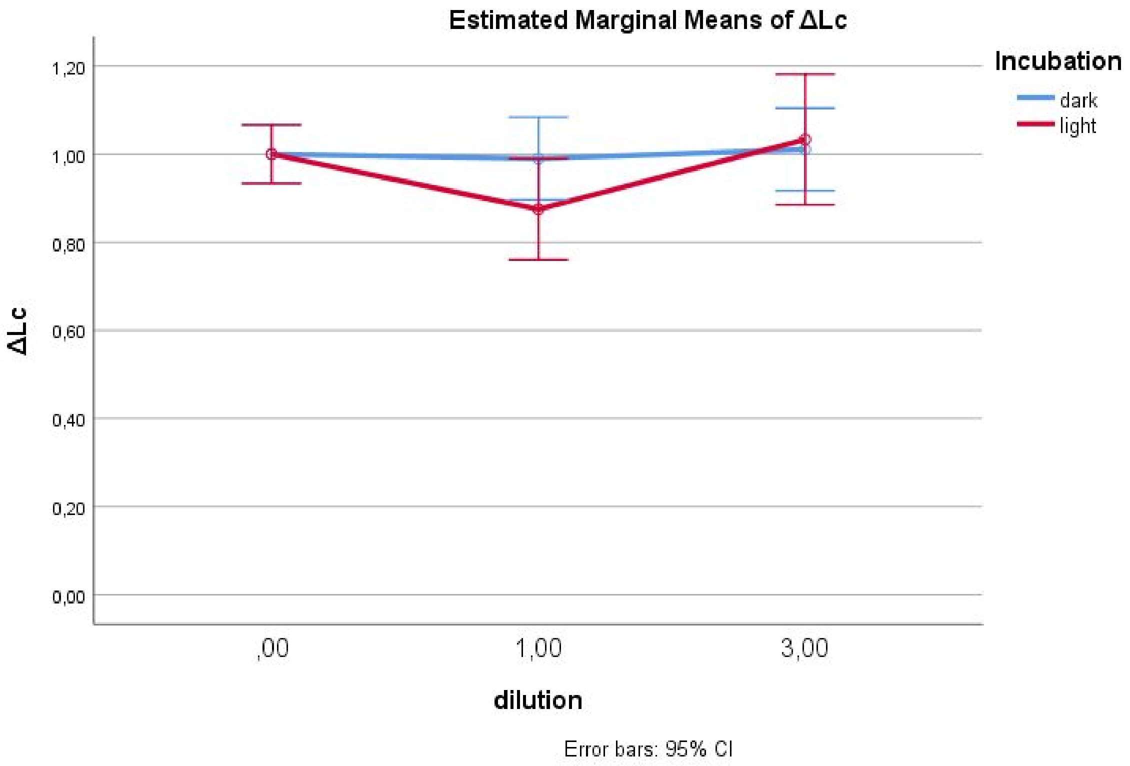

Figure S10 Comparison of ΔLc from light and dark incubations for PE
The figure shows the mean averages and 95% confidence intervals (CI) for sea-urchin larval length increase (ΔLc). The x-axis represents the 4 dilutions tested. Clearly overlapping CI´s indicate no significant difference between the light (pink) and dark (blue) incubation.

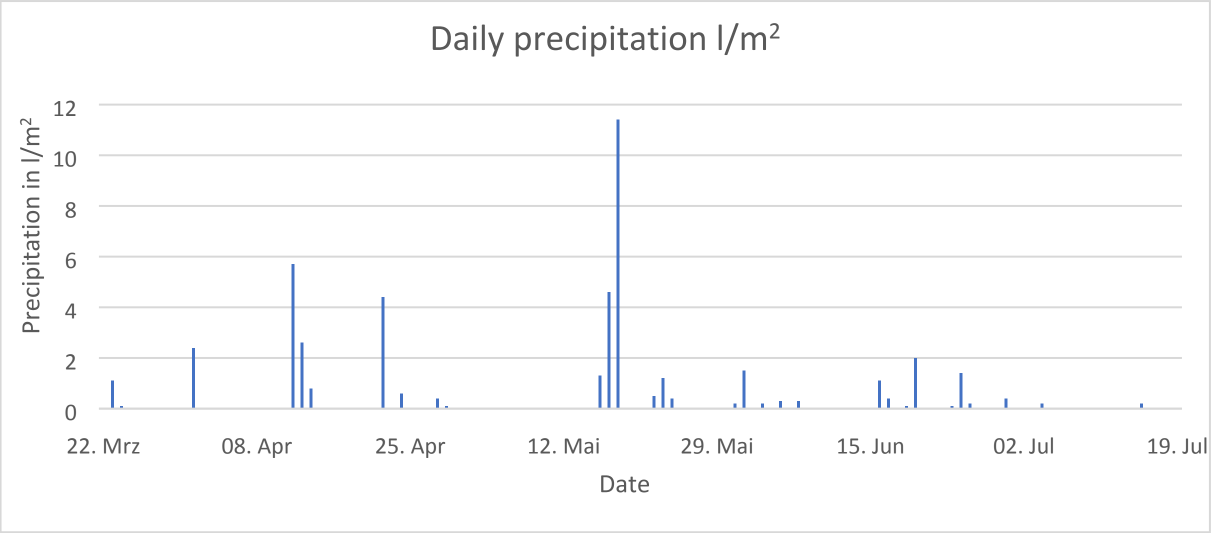

Figure S11 Daily precipitation as measured by the ECIMAT weather station of the Mesocosm2022. Note that heavy rain was experienced during the first 28 days (22.03 – 18.04) already. Unfortunately, weather data for this timeframe is partially missing due to malfunctions in the system.

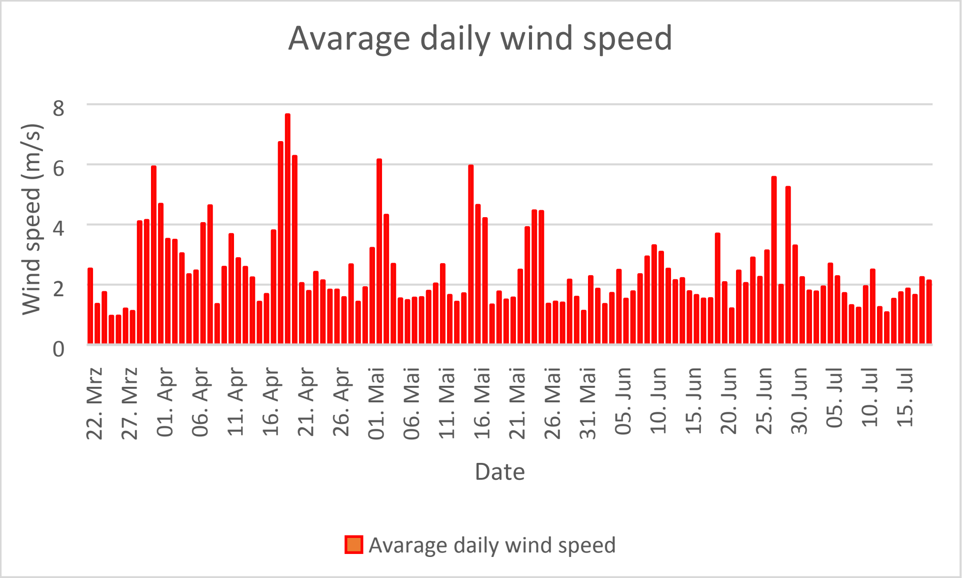

Figure S12 Wind speed (red) recordings of the Mesocosm2022. The wind speed is calculated as the daily average wind speed.
